# Enhancing oral competence: Evidence-based strategies for developing foreign language speaking skills in Deaf and Hard-of-Hearing (DHH) learners

**DOI:** 10.1371/journal.pone.0348196

**Published:** 2026-05-12

**Authors:** Izabela Olszak, Aleksandra Borowicz

**Affiliations:** 1 The John Paul II Catholic University of Lublin, Humanities, Applied Linguistics Department, Lublin, Poland; 2 Institute of Pedagogy, Department of Special Education, The John Paul II Catholic University of Lublin, Lublin, Poland; Father Muller Charitable Institutions, INDIA

## Abstract

This study examines evidence-based pedagogical strategies aimed at enhancing communication competence, with a particular focus on foreign language speaking skills, among Deaf and Hard of Hearing (DHH) students at the primary education level. Acknowledging the distinct linguistic, cognitive, and communicative characteristics of DHH learners, the research addresses the central question: Which instructional techniques most effectively promote the development of speaking and broader communicative abilities in this population? Employing a qualitative research design, the study engaged 55 primary-level DHH students and utilized classroom observations, student questionnaires, and semi-structured interviews with teachers and educational specialists, thereby adopting a multi-perspective methodological framework that enables the triangulation of learner, classroom and professional perspectives. The findings underscore the efficacy of multimodal and visually enriched teaching approaches, the value of differentiated, learner-centered instruction, and the importance of collaborative learning environments in supporting oral foreign language development. The research provides empirical insights into the design of inclusive and responsive language education practices for DHH learners, including those relevant to foreign language speaking skills, drawing on triangulated qualitative evidence from multiple educational stakeholders, contributing to the advancement of both academic achievement and communicative competence in diverse classroom settings.

## Introduction

Language acquisition is a critical foundation for both academic achievement and social integration among Deaf and Hard of Hearing (DHH) students. However, due to sensory, linguistic, and cognitive challenges, DHH learners frequently experience difficulties in developing and mastering oral communication and engaging in complex language use. These barriers can hinder their ability to access curriculum content, articulate ideas effectively, and participate in collaborative learning contexts. Accordingly, there is an urgent need to identify and implement evidence-based instructional strategies that accommodate the distinctive linguistic profiles of DHH learners and optimize their communicative competence.

Recent research has increasingly emphasized the importance of inclusive, multimodal pedagogies that integrate visual, gestural, and spoken modalities to address the diverse needs of DHH students. Effective teaching strategies must not only address the sensory limitations associated with hearing loss but also harness learners’ strengths in visual processing, sign language proficiency, and contextual inference. Despite advances in this field, systematic empirical investigation remains limited regarding which strategies most effectively foster oral language development and speaking skills, also in the context of foreign language learning, in primary-level DHH learners.

The present study addresses this gap by investigating the central question:

Which evidence-based techniques most effectively enhance speaking and communication skills in primary-level DHH students? Adopting a qualitative research design, the study engaged 55 primary-level DHH students through classroom observations and learner questionnaires, complemented by semi-structured interviews with teachers and educational specialists, thus integrating multiple sources of data to capture both learner experiences and instructional practices from different educational perspectives. This multi-perspective approach aims to generate empirically grounded recommendations for the design of inclusive, responsive, and linguistically enriched learning environments that promote both academic success and communicative competence in DHH learners.

## Literature review

### Teaching strategies used by educators

Language acquisition in deaf and hard-of-hearing (d/Deaf or d/DHH) individuals does not occur naturally and spontaneously as it does in hearing children. Instead, it requires intentional and systematic support. As Domagała-Zyśk [1] points out, “a deaf student must be *taught* language.” Teachers, parents, and specialists in speech therapy and special education play a crucial role in this process.

This section provides a review of the literature concerning best practices in teaching foreign languages to Deaf and Hard of Hearing (DHH) students. Due to the heterogeneity of this population, as well as the limited number of studies and empirically grounded publications, there are no universally accepted guidelines for foreign language instruction for this group. The literature includes case studies of individual DHH learners and results from studies conducted with small populations. These sources reveal recurring insights regarding promising methods for supporting bilingual deaf learners.

Studies in American educational literature identify several effective instructional practices across cognitive domains such as reading, writing, and speaking in a foreign language. For instance, Scott, Amadi, and Butts [2] conducted a review of research on DHH learners and identified three core instructional pillars that support the development of literacy skills in multilingual deaf and hard-of-hearing students:

a) explicit instruction – it involves deliberate, step-by-step teaching of specific skills (e.g., new vocabulary or expressions), with clearly defined lesson goals modelled and monitored by the teacher. Modelling, such as demonstrating how to understand or use a new word, allows students to grasp the word’s meaning and provides opportunities for repeated use across various contexts, which aids retention. This method is particularly useful for teaching high-utility and frequently recurring words, relevant across multiple disciplines [3]. Such an approach also facilitates the transfer of meaning from the first language to the second [4,5].
b) activation of background knowledge – learning becomes more effective when new information can be linked to what the learner already knows. Background knowledge refers to a student’s existing conceptual, experiential, and lexical framework, which provides context for understanding new content. Communication limitations may hinder access to this contextual knowledge, creating challenges in reading comprehension and vocabulary learning [6]. Strategies for activating background knowledge include recalling prior knowledge, referencing students’ lived experiences, and drawing on familiar social and cultural contexts. Supporting materials—such as videos, images, and other visuals—can be instrumental. This method has been shown to aid learners in completing cloze tests and similar language tasks [7].
c) metacognitive strategy instruction – several studies highlight the effectiveness of metacognitive strategies in reading and writing instruction. These include planning one’s approach to a task, monitoring comprehension, and evaluating the success of learning methods. Typical metacognitive actions include drawing connections between new and prior knowledge, checking understanding during reading, identifying key information, and making predictions based on available content [8]. For example, Brigham and Hartman [9] found that prediction strategies enhanced reading comprehension in d/Dhh students learning in total communication classrooms [2,10].

These techniques are especially effective for Deaf and Hard of Hearing (DHH) students because they compensate for limited access to spoken language, promote vocabulary acquisition, and foster strategies for continued language learning. These methods may be further enhanced through visual aids, sign language, translation, and other communication accommodations.

A collection of valuable recommendations for supporting deaf students in foreign language learning has been formulated by Polish researcher Ewa Domagała-Zyśk [1,11]. Drawing from her teaching experience with DHH students learning English, she identifies a range of effective practices grouped into the following functional categories:

a) environmental conditions – effective foreign language instruction for DHH students requires a learning environment that maximizes the use of available communication channels. Crucial are appropriate acoustic and lighting conditions that enable speechreading and observation of facial expressions and gestures. Technological aids, such as induction loops and FM systems, can provide additional support.
b) strategies for supporting spoken language perception through visual aids – In foreign language learning, the use of visual techniques is essential to support perception and speechreading. Effective strategies include the use of Polish and British Sign Language, as well as the International Phonetic Alphabet (IPA). The Cued Speech method [12] has shown promise in enhancing phonological input through visual articulation cues. Maintaining eye contact, clear articulation, and appropriately moderated facial expressions and gestures further contribute to comprehension. Visual aids, such as pictures, labels, and worksheets, as well as transcriptions, paraphrases, and explanations of complex terms, help gradually expand students’ vocabulary.
c) individualization of the instructional process, instructional effectiveness increases when the teaching process is tailored to individual learners’ needs. Individualization involves adjusting both the pace and mode of content delivery. Clear instructions, personal relevance, and contextualized learning materials support this process. Optimal results are often achieved in small groups (2–3 students) or ideally in one-on-one settings, allowing for customization of instruction.
d) emotional climate and motivational support – creating a safe and supportive emotional climate is essential for student success. Positive reinforcement, a focus on achievements, and teacher encouragement help build trust and sustain motivation. One of the most motivating factors for students is the opportunity to use the foreign language in authentic contexts—for example, in conversations with native speakers [1,11].

### Methodology – Research aim, participants, and teaching context

This research involving human subjects was approved by the Ethics Review Board of the Institute of Pedagogy at the John Paul II Catholic University of Lublin (approval number: IP/KE-8/2025). Written informed consent was obtained from all participants prior to their participation in the study.

The primary objective of this qualitative investigation was to address the following research question: *Which instructional techniques most effectively enhance foreign language speaking and communication skills in Deaf and Hard of Hearing (DHH) students?* This objective directly reflects the central aim of the study, namely, to identify evidence-based instructional strategies that support the development of speaking abilities and broader communicative competence among primary-level DHH learners. Participants responded to a structured in a foreign language questionnaire consisting of 22 items, organized into designated thematic blocks, with each item rated on a three-point frequency scale: ‘I never do,’ ‘I sometimes do,’ and ‘I always do’. The questionnaire was specifically designed to examine the frequency with which learners employ particular speaking-related learning strategies in a foreign language, allowing the researchers to identify patterns associated with more effective communicative behaviour. To complement these quantitative data, classroom observations and semi-structured interviews were conducted to obtain richer, contextualized insights into instructional practices and learner experiences. The integration of these data sources enabled a more comprehensive interpretation of students’ strategy use and the pedagogical contexts in which these strategies are encouraged or supported.

Fifteen primary schools serving DHH students were randomly approached to participate in the study. Ten schools from the Lublin, Greater Poland, and Masovian Voivodeships consented, and a snowball sampling strategy was employed to reach the targeted sample size. The final cohort comprised 55 students in grades VII and VIII, including 32 female and 23 male learners. In addition to demographic characteristics, information regarding participants’ hearing profiles was collected. The sample included students representing varying degrees of hearing loss (mild, moderate, severe, and profound), as well as differing levels of access to auditory support technologies. Many participants used amplification devices such as hearing aids or cochlear implants, and the duration of device use varied among learners depending on the timing of diagnosis and intervention. These variables were included in the statistical analysis because previous research indicates that both the degree of hearing loss and access to amplification technologies may significantly influence language acquisition and communicative behaviour in DHH learners. These students follow an adapted Core Curriculum for General Education, modified to address the specific linguistic and cognitive needs of individuals with hearing impairments. English language instruction in these settings incorporates specialized methodologies focused on speech development, vocabulary acquisition, and articulation enhancement, adapted to learners’ individual communication profiles to optimize outcomes. The study focused on English as a foreign language because foreign language instruction in Polish schools provides a structured context in which speaking strategies, pronunciation practice, and communicative interaction are systematically developed. For DHH learners, such contexts may highlight both the challenges and the compensatory strategies associated with spoken communication. The study was conducted between 13/01/2025 and 31/01/2025. The questionnaire was made available to teachers to administer in order to obtain the most reliable results and to avoid causing emotional discomfort among the participants in connection with their difficulties and functional challenges. This procedure served an important methodological purpose: by involving teachers, professionals familiar with the students’ needs, behaviours, and communication patterns, the data collection process could be carried out in a supportive and ethically responsible manner. Moreover, delegating the administration of the survey to trusted educators minimized the risk of response bias, reduced anxiety among respondents, and ensured that the environment in which the data were collected remained stable and non-intrusive. Such an approach is consistent with best practices in research involving vulnerable populations, where safeguarding participants’ well-being is as crucial as maintaining the validity and reliability of the findings, while also ensuring that the data gathered through questionnaires, observations, and interviews collectively provide a comprehensive and context-sensitive understanding of students’ communicative strategy use.

The questionnaire instrument consisted of several thematic sections addressing key dimensions of foreign language speaking strategy use. These sections included pronunciation and articulation strategies, sentence construction and foreign language grammatical formulation, conversational interaction strategies, fluency development techniques, strategies for seeking clarification or assistance during communication, and strategies supporting vocabulary retrieval and expression. Each item was formulated as a behavioural statement describing a specific communicative action (e.g., repeating difficult words, asking for clarification, or using visual cues to support speech). Students indicated how frequently they used each strategy using the three-point response scale described above. The thematic grouping of items allowed the researchers to analyse patterns of strategy use across different dimensions of speaking competence.

DHH learners represent a highly heterogeneous population, with variation in the nature and degree of hearing loss, the timing of onset (prelingual versus postlingual), and the access to and efficacy of technological or rehabilitative support. These factors significantly influence linguistic development, including foreign language acquisition [2,13–18]. Consequently, foreign language instruction should avoid a uniform approach, instead prioritising individualised strategies grounded in thorough diagnostic assessment of each learner’s language competence. Such assessment, encompassing both the first language and prior foreign language exposure, is critical for tailoring instruction, calibrating difficulty levels, and preventing the frustration that can undermine motivation and engagement.

Frustration arising from materials that exceed a learner’s capabilities can diminish motivation, foster feelings of helplessness, and lead to disengagement. Therefore, individualized instruction should extend beyond content selection and methodological approaches to include the establishment of clear, realistic, and personally meaningful learning objectives [19]. Goals such as achieving certification, preparing for examinations, or developing specific communicative competencies can enhance intrinsic motivation, strengthen learner autonomy, and support sustained engagement.

In the Polish context, DHH education faces complex systemic challenges. The scarcity of specialized schools, predominantly concentrated in urban areas, limits access for students in rural regions. Although inclusive education policies seek to integrate DHH learners into mainstream classrooms, many schools lack the requisite infrastructure, including trained educators, interpreters, and assistive technologies. This often results in substantial obstacles to effective learning.

Communication barriers are further compounded by the limited application of Polish Sign Language (PSL) in education. Despite its official recognition, PSL remains underutilized due to insufficient teacher proficiency, a lack of appropriate instructional materials, and inadequate training in inclusive pedagogy and the developmental needs of DHH learners. The deficit of specialized support staff, including interpreters, speech therapists, and special education counsellors, further exacerbates these challenges. While Polish legislation guarantees the right to education for DHH students, inconsistencies in enforcement, insufficient funding for accommodations, and regional disparities continue to hinder equitable access to quality education.

The inclusion of this contextual discussion is intended to clarify the broader educational environment in which the participating students learn and communicate, as systemic factors related to language access, communication modalities, and institutional resources may indirectly influence the development and use of speaking strategies among DHH learners. With regard to the statistical procedures applied in the subsequent analysis, non-parametric tests were selected due to the relatively small sample size and the ordinal nature of the questionnaire responses. Specifically, the Mann–Whitney U test was used to compare differences between two independent groups, while the Kruskal–Wallis test was applied when comparing more than two groups across variables such as degree of hearing loss, onset of hearing impairment, and duration of amplification use. These tests are widely recommended in educational and social science research when the assumptions required for parametric tests, such as normal distribution and interval-level measurement, cannot be confidently satisfied. The use of these procedures, therefore ensured that the statistical analysis remained methodologically appropriate for the structure and scale of the collected data.

## Results of the study

The study analysis evaluated the significance of differences using statistical methods. The grouping variables considered were gender, degree of hearing loss, method of communication with others, and the onset time of hearing loss. To conduct the analysis, the Mann-Whitney U test was employed for independent samples when the grouping variable divided participants into two categories, while the Kruskal-Wallis test was used when participants were divided into more than two groups. A significance level of p = 0.05 was established as the threshold. All results identified as statistically significant are marked in red within the presented tables.

### III. 1. Speaking learning strategies in a foreign language, grouping variable: gender

#### Mann-Whitney U test for independent samples.

The Mann-Whitney U test was conducted to examine whether the use of speaking learning strategies differed between male and female participants (see Table 1). Overall, the comparison did not reveal statistically significant differences (U = 514.00, Z = 0.36, p = 0.716, r = 0.04, 95% CI [−0.18, 0.26]).

**Table 1:** Speaking learning strategies, grouping variable: gender.

Null hypothesis	Significance
I search for a synonym	.731
I try to pronounce it quietly so that no one hears	.878
I ask the person I am talking to how to say this word	.786
I try my best to say this word with a hope that people will understand me	.984
I try to write it down to put it together	.599
I try to give up what I wanted to say	.634
I try to say it in a different way	.429
I use DeepL, Google Translator, chatGPT, or other translation app	.715
I search for a synonym	.881
I explain/describe the word with the hope that the person I am talking to will know	.688
I use DeepL, Google Translator, chatGPT, or other translation (apps)	.926
I try to give up what I wanted to say	.848
I focus on fluency rather than worrying about mistakes	.541
I am thinking about how bad my pronunciation and grammar are	.637
I actively seek feedback on my pronunciation and grammar	.820
I prepare a list of words/discussion topics to help myself in conversation	.803
I try to speak as little as possible	.709
I try to incorporate new words	.571
I make an effort to contribute to the conversation to practise my language skills	.548
I write new words which I hear to review/use later	.621
I use the words in sentences to understand their context	.573
I seek clarification from people	.879
I practise using the rule in different sentences	.416
I create flashcards to remember the words:	.764

The results were analysed in 6 category blocks of the speaking strategies related to:

1^st^ block: pronunciation and vocabulary

2^nd^ block: sentence construction and rephrasing

3^rd^ block: translation tools and finding synonyms

4^th^ block: fluency, feedback, and interaction during speaking practice

5^th^ block: communication in group settings, such as preparation and contribution

6^th^ block: clarification, rule practice, and grammar challenges

Across all six blocks, there were no statistically significant differences between genders (all p-values > 0.05), indicating that both male and female students employ speaking strategies with similar frequency. Both groups are equally likely to employ strategies like searching for synonyms, asking for clarification, attempting to pronounce unfamiliar words, writing down words, giving up, paraphrasing, using translation tools, synonyms, descriptions, reliance on translation tools, focusing on fluency, worrying about mistakes, seeking feedback, preparing discussion lists, seeking clarification, practicing rules, and creating flashcards. The detailed results of the frequency of using speaking strategies within the 6 blocks are as follows:

**Block 1 – Pronunciation and vocabulary**

- Q1: I try my best to say this word so that people will understand me (p = 0.984)
- Q2: I try to pronounce it quietly so that no one hears (p = 0.878)
- Q3: I ask the person I am talking to how to say this word (p = 0.786)

**Block 2 – Sentence construction and rephrasing**

- Q4: I try to say it in a different way (p = 0.429)
- Q5: I try to give up what I wanted to say (p = 0.634)
- Q6: I try to write it down to put it together (p = 0.599)
- Q7: I use DeepL, Google Translator, ChatGPT, or other translation apps (p = 0.715)

**Block 3 – Translation tools and finding synonyms**

- Q8: I search for a synonym (p = 0.881)
- Q9: I explain/describe the word with the hope that the person I am talking to will know (p = 0.688)
- Q10: I use translation apps (p = 0.926)
- Q11: I try to give up what I wanted to say (p = 0.848)

**Block 4 – Fluency, feedback, and interaction**

- Q12: I focus on fluency rather than worrying about mistakes (p = 0.541)
- Q13: I am thinking about how bad my pronunciation and grammar are (p = 0.637)
- Q14: I actively seek feedback on my pronunciation and grammar (p = 0.820)

**Block 5 – Communication in group settings**

- Q15: I prepare a list of words/discussion topics to help myself in conversation (p = 0.803)
- Q16: I write new words which I hear to review/use later (p = 0.621)
- Q17: I try to incorporate new words (p = 0.571)
- Q18: I make an effort to contribute to the conversation to practice my language skills (p = 0.548)

**Block 6 – Clarification, rule practice and grammar challenges**

- Q19: I seek clarification from people (p = 0.879)
- Q20: I practice using the rule in different sentences (p = 0.416)
- Q21: I create flashcards to remember the words (p = 0.764)
- Q22: I use the words in sentences to understand their context (p = 0.573)

### III. 2 Speaking learning strategies in a foreign language, grouping variable: degree of hearing loss

#### Kruskal-Wallis test for independent samples.

The Kruskal-Wallis test results (see Table 2) indicate that most speaking learning strategies are employed similarly across participants with different degrees of hearing loss. Most strategies showed no significant difference across groups, with the exception of one vocabulary-related strategy.

**Table 2:** Speaking learning strategies, grouping variable: degree of hearing loss.

Null hypothesis	Significance
I search for a synonym	.495
I try to pronounce it quietly so that no one hears	.445
I ask the person I am talking to how to say this word	.864
I try my best to say this word with the hope that people will understand me	.348
I try to write it down to put it together	.640
I try to give up what I wanted to say	.920
I try to say it in a different way	.720
I use DeepL, Google Translator, chatGPT, or other translation app	.159
**I search for a synonym**	**.020**
I explain/describe the word with the hope that the person I am talking to will know	.375
I use DeepL, Google Translator, chatGPT, or other translation (apps)	.109
I try to give up what I wanted to say	.123
I focus on fluency rather than worrying about mistakes	.555
I am thinking about how bad my pronunciation and grammar are	.283
I actively seek feedback on my pronunciation and grammar	.443
I prepare a list of words/discussion topics to help myself in conversation	.348
I try to speak as little as possible	.649
I try to incorporate new words	.703
I make an effort to contribute to the conversation to practise my language skills	.430
I write new words which I hear to review/use later	.896
I use the words in sentences to understand their context	.657
I seek clarification from people	.500
I practise using the rule in different sentences	.923
I create flashcards to remember the words:	.949

Specifically, Q8: *“I search for a synonym”* demonstrated a statistically significant difference between groups (p = 0.020, r = 0.31, 95% CI [0.05, 0.53]), indicating a moderate effect size. This finding suggests that learners with different degrees of hearing loss may employ different approaches when attempting to overcome vocabulary gaps during speaking activities.

The applied speaking strategies within the 6 blocks are as follows:

**Block 1 – Pronunciation and vocabulary**

- Q1: I try my best to say this word (p = 0.348)
- Q2: I try to pronounce it quietly (p = 0.445)
- Q3: I ask the person I am talking to how to say this word (p = 0.864)
- Q8: I search for a synonym (p = 0.020, r = 0.31, 95% CI [0.05, 0.53]) statistically significant

**Block 2 – Sentence construction and rephrasing**

- Q4: I try to say it in a different way (p = 0.720)
- Q5: I try to give up what I wanted to say (p = 0.920)
- Q6: I try to write it down to put it together (p = 0.640)
- Q7: I use DeepL, Google Translator, ChatGPT, or other translation apps (p = 0.159)

**Block 3 – Translation tools and finding synonyms**

- Q9: I explain/describe the word (p = 0.375)
- Q10: I try to give up (p = 0.123)
- Q11: I use translation apps (p = 0.109)

**Blocks 4–6 – Fluency, feedback, group communication and clarification**

- Q12–Q22 (all p > 0.28) – non-significant

### III.3. Speaking learning strategies in a foreign language, grouping variable: method of communication with the environment

#### Kruskal-Wallis test for independent samples.

The Kruskal–Wallis test was conducted to examine whether the use of speaking learning strategies differed depending on the method of communication with the environment (see Table 3). Effect sizes were calculated using Cramér’s V and 95% confidence intervals were estimated to assess the strength and precision of the observed associations. The majority of strategies did not differ significantly between communication groups (all p > 0.05). However, statistically significant differences were observed for two strategies.

**Table 3:** Speaking learning strategies, grouping variable: method of communication with the environment.

Null hypothesis	Significance
I search for a synonym	.319
I try to pronounce it quietly so that no one hears	.795
I ask the person I am talking to how to say this word	.678
I try my best to say this word with the hope that people will understand me	.513
I try to write it down to put it together	.461
I try to give up what I wanted to say	.766
I try to say it in a different way	.804
I use DeepL, Google Translator, chatGPT or other translation app	.176
**I search for a synonym**	**.009**
I explain/describe the word with the hope that the person I am talking to will know	.168
I use DeepL, Google Translator, chatGPT or other translation (apps)	.167
I try to give up what I wanted to say	.056
I focus on fluency rather than worrying about mistakes	.448
I am thinking how bad my pronunciation and grammar is	.235
I actively seek feedback on my pronunciation and grammar	.433
I prepare a list of words/discussion topics to help myself in conversation	.386
I try to speak as little as possible	.321
I try to incorporate new words	.613
I make an effort to contribute to the conversation to practise my language skills	.522
I write new words which I hear to review/use later	.939
I use the words in sentences to understand their context	.808
I seek clarification from people	.307
I practise using the rule in different sentences	.776
I create flashcards to remember the words:	.794

The detailed speaking strategy results within the six blocks are as follows:

**Block 1 – Pronunciation and vocabulary**

- Q2: I try to pronounce it quietly so that no one hears (p = 0.795)
- Q3: I ask the person I am talking to how to say this word (p = 0.678)
- Q1: I try my best to say this word with the hope that people will understand me (p = 0.513)
- Q8: I search for a synonym (p = 0.319)

**Block 2 – Sentence construction and rephrasing**

- Q4: I try to say it in a different way (p = 0.804)
- Q5: I try to give up what I wanted to say (p = 0.766)
- Q6: I try to write it down to put it together (p = 0.461)
- Q7: I use DeepL, Google Translator, ChatGPT, or other translation apps (p = 0.176)

**Block 3 – Translation tools and finding synonyms**

- Q9: I explain/describe the word with the hope that the person I am talking to will know (p = 0.168)
- Q11: I use DeepL, Google Translator, ChatGPT, or other translation apps (p = 0.167)
- Q10: I try to give up what I wanted to say (p = 0.056)
**Q8: I search for a synonym (p = 0.009, V = 0.30, 95% CI [0.03, 0.50]) – statistically significant; participants’ method of communication influences use of synonyms with a moderate effect size)

**Block 4 – Fluency, feedback, and interaction during speaking practice**

- Q12: I focus on fluency rather than worrying about mistakes (p = 0.448)
- Q14: I actively seek feedback on my pronunciation and grammar (p = 0.433)
- Q15: I prepare a list of words/discussion topics to help myself in conversation (p = 0.386)
- Q13: I am thinking about how bad my pronunciation and grammar is (p = 0.235)

**Block 5 – Communication in group settings (preparation and contribution)**

- Q16: I write new words that I hear to review/use later (p = 0.939)
- Q17: I try to incorporate new words (p = 0.613)
- Q18: I make an effort to contribute to the conversation to practise my language skills (p = 0.522)
- Q15: I prepare a list of words/discussion topics to help myself in conversation (p = 0.321)

**Block 6 – Clarification, rule practice and grammar challenges**

- Q22: I use the words in sentences to understand their context (p = 0.808)
- Q21: I create flashcards to remember the words (p = 0.794)
- Q20: I practise using the rule in different sentences (p = 0.776)
- Q19: I seek clarification from people (p = 0.307)

### III. 4. Speaking learning strategies in a foreign language, grouping variable: time of onset of hearing loss

#### Mann-Whitney U test for independent samples.

The Mann-Whitney U test was conducted to examine whether the use of speaking learning strategies differed depending on whether learners had prelingual or postlingual hearing loss (see Table 4). Effect sizes were calculated using Cramér’s V and 95% confidence intervals were estimated to assess the strength and precision of the observed associations. Most strategies did not differ significantly between groups (all p > 0.05). However, several strategies showed statistically significant differences, suggesting that time of onset of hearing loss can influence specific speaking strategies.

**Table 4:** Speaking learning strategies, grouping variable: time of onset of hearing loss.

Null hypothesis	Significance
I search for a synonym	.678
I try to pronounce it quietly so that no one hears	.258
I ask the person I am talking to how to say this word	.894
I try my best to say this word with the hope that people will understand me	.468
I try to write it down to put it together	.852
I try to give up what I wanted to say	.689
I try to say it in a different way	.849
I use DeepL, Google Translator, chatGPT, or other translation app	.121
I search for a synonym	.751
I explain/describe the word with the hope that the person I am talking to will know	.275
I use DeepL, Google Translator, chatGPT, or other translation (apps)	.202
I try to give up what I wanted to say	.294
I focus on fluency rather than worrying about mistakes	.273
I am thinking about how bad my pronunciation and grammar are	.061
I actively seek feedback on my pronunciation and grammar	.403
I prepare a list of words/discussion topics to help myself in conversation	.621
I try to speak as little as possible	.902
I try to incorporate new words	.206
I make an effort to contribute to the conversation to practice my language skills	.256
**I write new words which I hear to review/use later**	**.045**
I use the words in sentences to understand their context	.683
**I seek clarification from people**	**.039**
I practice using the rule in different sentences	.954
I create flashcards to remember the words:	.948

The detailed results within the six blocks are presented below:

**Block 1 – Pronunciation and vocabulary**

- Q3: I ask the person I am talking to how to say this word (p = 0.894)
- Q8: I search for a synonym (p = 0.678)
- Q1: I try my best to say this word with the hope that people will understand me (p = 0.468)
- Q2: I try to pronounce it quietly so that no one hears (p = 0.258)

**Block 2 – Sentence construction and rephrasing**

- Q6: I try to write it down to put it together (p = 0.852)
- Q4: I try to say it in a different way (p = 0.849)
- Q5: I try to give up what I wanted to say (p = 0.689)
- Q7: I use DeepL, Google Translator, ChatGPT, or other translation apps (p = 0.121)

**Block 3 – Translation tools and finding synonyms**

- Q8: I search for a synonym (p = 0.751)
- Q10: I try to give up what I wanted to say (p = 0.294)
- Q9: I explain/describe the word with the hope that the person I am talking to will know (p = 0.275)
- Q11: I use DeepL, Google Translator, ChatGPT, or other translation apps (p = 0.202)

**Block 4 – Fluency, feedback and interaction during speaking practice**

- Q15: I prepare a list of words/discussion topics to help myself in conversation (p = 0.621)
- Q14: I actively seek feedback on my pronunciation and grammar (p = 0.403)
- Q12: I focus on fluency rather than worrying about mistakes (p = 0.273)
- Q13: I am thinking about how bad my pronunciation and grammar is (p = 0.061)

**Block 5 – Communication in group settings (preparation and contribution)**

- Q15: I prepare a list of words/discussion topics to help myself in conversation (p = 0.902)
- Q18: I make an effort to contribute to the conversation to practice my language skills (p = 0.256)
- Q17: I try to incorporate new words (p = 0.206)
**Q16: I write new words which I hear to review/use later (p = 0.045, V = 0.28, 95% CI [0.01, 0.50]); prelingual and postlingual learners differ in vocabulary recording strategy.p = 0.045) – statistically significant difference

**Block 6 – Clarification, rule practice and grammar challenges**

- Q20: I practice using the rule in different sentences (p = 0.954)
- Q21: I create flashcards to remember the words (p = 0.948)
- Q22: I use the words in sentences to understand their context (p = 0.683)
**Q19: I seek clarification from people (p = 0.039, V = 0.29, 95% CI [0.02, 0.51]) – statistically significant; prelingual and postlingual learners differ in reliance on clarification during communication.

## Conclusion

This study offers a comprehensive, multi-perspective view on effective foreign language instruction for Deaf and Hard of Hearing (DHH) students, responding directly to the research questions posed.

In addressing speaking and communication skills in a foreign language, findings suggest that while most DHH students utilize similar strategies, such as rephrasing, rehearsing speech silently, or relying on translation tools, specific strategies, particularly those involving vocabulary exploration and interpersonal clarification, vary depending on the degree of hearing loss, time of onset, and communication method. Fluency-enhancing strategies, including practice with peer feedback and real-time captioning support, were widely used and valued across all participant groups.

An important cross-cutting theme is the critical role of visualisation, metalinguistic awareness, and multimodal support in enhancing foreign language acquisition. As noted, the study by Berent et al. [20] underscores the potential of the *Focus on Form* (FonF) approach in drawing learners’ attention to linguistically significant forms within meaningful communication. Although developed for hearing learners, FonF proves effective for DHH students, particularly when adapted to leverage their dominant visual modality. Techniques such as input enhancement and implicit feedback align with this approach and support both grammatical development and strategic language processing [21].

This study offers a multi-perspective analysis of speaking strategies among DHH students, revealing that:

- Most strategies (rephrasing, rehearsing silently, using translation tools) are widely shared across gender, hearing loss, and communication methods.
- Vocabulary strategies (searching for synonyms) and interpersonal clarification strategies vary depending on the degree and onset of hearing loss, and communication method, highlighting the need for tailored interventions.
- Fluency-enhancing strategies, feedback practices, and visual supports are universally valuable.

Although only a limited number of statistically significant differences were identified, the findings provide valuable insight into the speaking strategies employed by DHH learners. The predominance of non-significant differences suggests that many communication strategies are widely shared across learners regardless of demographic variables. However, the observed differences related to the onset of hearing loss indicate that certain strategies, particularly those involving clarification and vocabulary recording, may require more individualized pedagogical support. In summary, successful foreign language instruction for DHH learners necessitates a multimodal, differentiated and contextually situated approach. It should be noted that the absence of statistically significant differences in most strategy categories does not imply that the groups are identical, but rather that no reliable differences were detected within the present sample. Pedagogical strategies that combine form-focused instruction with visually enriched and collaborative learning settings demonstrate particular efficacy. These findings provide a basis for tailored, evidence-based interventions and support the advancement of inclusive educational practices across varied learning environments.

## Supporting information

S1 File Questionnaire. Strategies for developing reading comprehension, vocabulary and writing skills among Deaf and Hard-of-Hearing Students. (DOCX)

S2 File Excel database. Excel database containing the obtained data for analysis. (XLSX)
