# Supplementary material for: Enhancing oral competence: Evidence-based strategies for developing foreign language speaking skills in Deaf and Hard-of-Hearing (DHH) learners
Source: PLoS One. 2026 May 12;21(5):e0348196. doi: 10.1371/journal.pone.0348196 (PMC13166900; doi:10.1371/journal.pone.0348196)
Supplement: S1 File — (DOCX) [file pone.0348196.s001.docx]

**QUESTIONNAIRE ON STRATEGIES FOR DEVELOPING**

**READING COMPREHENSION, VOCABULARY, AND WRITING SKILLS**

**AMONG DEAF AND HARD-OF-HEARING STUDENTS**

We kindly request you to complete the following questionnaire, which aims to examine the strategies used to develop reading comprehension, vocabulary, and writing skills among deaf and hard-of-hearing students. The research findings may provide significant scientific value, as they could shed light on the current state of foreign language acquisition within the Polish educational system. We guarantee full anonymity for all respondents, with no risk of identification.

PLEASE SELECT ONE RESPONSE:

*I don't do this. I sometimes do this. I always do this*.

1. **GENERAL INFORMATION**

**1. GENDER Female Male**

**2. DEGREE OF HEARING LOSS:**

**mild moderate severe profound**

**3. METHOD OF COMMUNICATION: ………………………………………….**

**4. ONSET OF HEARING LOSS: ……………………………………………………………….**

**5. NATURE OF HEARING LOSS: CONGENITAL ACQUIRED**

1. **Reading Strategies. Which ones do you use?**

| Instructions: For each statement, please indicate your response as follows:  *I don't do this. I sometimes do this. I always do this* |
| --- |

1. I try to guess what the text is about before reading it.

*I don't do this. I sometimes do this. I always do this*.

1. I begin by skimming the text, noting its length, structure, and visual aids such as graphs and photos.

*I don't do this. I sometimes do this. I always do this*.

1. I decide what to read in detail and what to skip.

*I don't do this. I sometimes do this. I always do this*.

1. To enhance my comprehension, I utilize the tables, drawings, and images provided in the text.

*I don't do this. I sometimes do this. I always do this*.

1. I pay attention to typographic cues, such as bolded words, to help me identify key information.

*I don't do this. I sometimes do this. I always do this*.

1. I adjust my reading speed depending on what I am reading.

*I don't do this. I sometimes do this. I always do this*.

1. I pause periodically to reflect on what I am reading.

*I don't do this. I sometimes do this. I always do this*.

1. I try to visualize or form mental images of the information in the text to help me remember what I read.

*I don't do this. I sometimes do this. I always do this*.

1. I try to guess the meaning of unknown words.

*I don't do this. I sometimes do this. I always do this*.

1. I take notes while reading to help me remember what I read.

*I don't do this. I sometimes do this. I always do this*.

1. I summarize what I have read to reflect on the key information in the text.

*I don't do this. I sometimes do this. I always do this*.

1. I underline or highlight information in the text to help me remember it better.

*I don't do this. I sometimes do this. I always do this*.

1. I paraphrase the text in my own words to better understand what I am reading.

*I don't do this. I sometimes do this. I always do this*.

1. I discuss what I am reading with others to ensure I understand the text.

*I don't do this. I sometimes do this. I always do this*.

1. **Vocabulary Learning Strategies: Which ones do you use?**

| Instructions: For each statement, please indicate your response as follows:  *I don't do this. I sometimes do this. I always do this*. |
| --- |

**When learning new English vocabulary, ….**

1 … I note down all words in a list, including their translations.

*I don't do this. I sometimes do this. I always do this*.

2. … I create mind maps with new words.

*I don't do this. I sometimes do this. I always do this*.

3. … I create flashcards with new words and their translations on the reverse side.

*I don't do this. I sometimes do this. I always do this*.

4. … I create vocabulary lists in digital applications (such as Quizlet).

*I don't do this. I sometimes do this. I always do this*.

5. … I write words on sticky notes and place them around my room or house.

*I don't do this. I sometimes do this. I always do this*.

6. … I try to associate new words with images or visual representations.

*I don't do this. I sometimes do this. I always do this*.

7. … I try to use new words in sentences to see them in context.

*I don't do this. I sometimes do this. I always do this*.

8. … I read through my vocabulary lists multiple times from top to bottom.

*I don't do this. I sometimes do this. I always do this*.

9. … I consistently utilize digital vocabulary applications (such as Quizlet) during daily activities (e.g., while commuting or every morning while having breakfast)

*I don't do this. I sometimes do this. I always do this*.

10. … I ask other people (e.g., my mother, sister, or a friend) to quiz me on new vocabulary.

*I don't do this. I sometimes do this. I always do this*.

1. **Writing Strategies: Which ones do you use?**

| Instructions: For each statement, please indicate your response as follows:  *I don't do this. I sometimes do this. I always do this*. |
| --- |

1. I discuss my ideas for writing in English with friends or family.

*I don't do this. I sometimes do this. I always do this*.

1. I create diagrams or visual aids to plan my English essays.

*I don't do this. I sometimes do this. I always do this*.

1. Ideas for writing in English occur to me while engaging in physical activities, such as walking.

*I don't do this. I sometimes do this. I always do this*.

1. I write about my personal life experiences in English.

*I don't do this. I sometimes do this. I always do this*.

1. I collaborate with other students on projects aimed at developing English writing skills.

*I don't do this. I sometimes do this. I always do this*.

1. I draw inspiration for my writing from listening to speeches or lectures in English.

*I don't do this. I sometimes do this. I always do this*.

1. Before I begin writing in English, I create a step-by-step plan.

*I don't do this. I sometimes do this. I always do this*.

1. I write in English about things I observe in nature or in my daily life.

*I don't do this. I sometimes do this. I always do this*.

1. I write short stories or poems in English.

*I don't do this. I sometimes do this. I always do this*.

1. When writing in English, I reflect deeply on my life.

*I don't do this. I sometimes do this. I always do this*.

1. I use computer software or applications that assist me with my English writing.

*I don't do this. I sometimes do this. I always do this*.

1. I include numerous examples and facts in my English essays.

*I don't do this. I sometimes do this. I always do this*.

1. I prefer English tasks with clear and precise instructions.

*I don't do this. I sometimes do this. I always do this*.

1. I enjoy English tasks that allow me to express my creativity.

*I don't do this. I sometimes do this. I always do this*.

1. **Speaking Learning Strategies - which ones do you use?**

**Instructions:**

**For each statement, please indicate your response as follows**

*I don't do this. I sometimes do this. I always do this*.

When I don’t know how to pronounce a particular word:

1. I search for a synonym.

*I don't do this. I sometimes do this. I always do this*.

1. I try to pronounce it quietly so that no one hears.

*I don't do this. I sometimes do this. I always do this*.

1. I ask the person I am talking to how to say this word.

*I don't do this. I sometimes do this. I always do this*.

1. I try my best to say this word with a hope that people will understand me.

*I don't do this. I sometimes do this. I always do this*.

When I don’t know how to say a particular sentence:

1. I try to write it down to put it together.

*I don't do this. I sometimes do this. I always do this*.

1. I try to give up what I wanted to say.

*I don't do this. I sometimes do this. I always do this*.

1. I try to say it in a different way.

*I don't do this. I sometimes do this. I always do this*.

1. I use DeepL, Google Translator, chatGPT, or other translation app.

*I don't do this. I sometimes do this. I always do this*.

When I don’t know how to translate a word into English:

1. I search for a synonym.

*I don't do this. I sometimes do this. I always do this*.

1. I explain/describe the word with the hope that the person I am talking to will know.

*I don't do this. I sometimes do this. I always do this*.

1. I use DeepL, Google Translator, chatGPT, or other translation (apps)

*I don't do this. I sometimes do this. I always do this*.

1. I try to give up what I wanted to say

*I don't do this. I sometimes do this. I always do this*.

When practicing speaking with a partner:

1. I focus on fluency rather than worrying about mistakes

*I don't do this. I sometimes do this. I always do this*.

1. I am thinking about how bad my pronunciation and grammar are.

*I don't do this. I sometimes do this. I always do this*.

1. I actively seek feedback on my pronunciation and grammar.

*I don't do this. I sometimes do this. I always do this*.

1. I prepare a list of words/discussion topics to help myself in conversation.

*I don't do this. I sometimes do this. I always do this*.

When participating in group discussions:

1. I try to speak as little as possible.

*I don't do this. I sometimes do this. I always do this*.

1. I try to incorporate new words.

*I don't do this. I sometimes do this. I always do this*.

1. I make an effort to contribute to the conversation to practise my language skills.

*I don't do this. I sometimes do this. I always do this*.

1. I write new words which I hear to review/use later.

*I don't do this. I sometimes do this. I always do this*.

W obliczu trudnego słowa lub reguły gramatycznej:

1. I use the words in sentences to understand their context.

*I don't do this. I sometimes do this. I always do this*.

1. I seek clarification from people.

*I don't do this. I sometimes do this. I always do this*.

1. I practise using the rule in different sentences.

*I don't do this. I sometimes do this. I always do this*.

1. I create flashcards to remember the words*.*

*I don't do this. I sometimes do this. I always do this*.
